# Studies on *Trueperella pyogenes* isolated from an okapi (*Okapia johnstoni*) and a royal python (*Python regius*)

**DOI:** 10.1186/s12917-020-02508-y

**Published:** 2020-08-14

**Authors:** Marwa F. E. Ahmed, Mazen Alssahen, Christoph Lämmler, Tobias Eisenberg, Madeleine Plötz, Amir Abdulmawjood

**Affiliations:** 1 Hygiene and Zoonoses Department, Faculty of Veterinary Medicine, Mansoura University, Elgomhoria Street 60, 35516 Mansoura, Egypt; 2 Institut für Hygiene und Infektionskrankheiten der Tiere, Justus-Liebig-Universität Gießen, Frankfurterstraße 85-91, D-35392 Gießen, Germany; 3 Landesbetrieb Hessisches Landeslabor (LHL), Schubertstraße 60, D-35392 Gießen, Germany; 4 Institute of Food Quality and Food Safety, Research Center for Emerging Infections and Zoonoses (RIZ), University of Veterinary Medicine Hannover, Bischofsholer Damm 15, D-30173 Hannover, Germany

**Keywords:** 16S rRNA gene, 16S-23S rDNA intergenic spacer region, LAMP, MALDI-TOF MS, pyolysin, *rpo*B, *tuf*

## Abstract

**Background:**

The present study was designed to characterize phenotypically and genotypically two *Trueperella pyogenes* strains isolated from an okapi (*Okapia johnstoni*) and a royal python (*Python regius*).

**Case presentation:**

The species identity could be confirmed by phenotypic properties, by MALDI-TOF MS analysis and by detection of *T. pyogenes* chaperonin-encoding gene *cpn*60 with a previously developed loop-mediated isothermal amplification (LAMP) assay. Furthermore, sequencing of the 16S ribosomal RNA (rRNA) gene, the 16S-23S rDNA intergenic spacer region (ISR), the target genes *rpo*B encoding the β-subunit of bacterial RNA polymerase, *tuf* encoding elongation factor tu and *plo* encoding the putative virulence factor pyolysin allowed the identification of both *T. pyogenes* isolates at species level.

**Conclusions:**

Both strains could be clearly identified as *T. pyogenes*. The *T. pyogenes* strain isolated in high number from the vaginal discharge of an okapi seems to be of importance for the infectious process; the *T. pyogenes* strain from the royal python could be isolated from an apparently non-infectious process. However, both strains represent the first isolation of *T. pyogenes* from these animal species.

## Background

*Trueperella pyogenes* is worldwide considered as part of the commensal biota of skin and mucous membranes of the upper respiratory and urogenital tract of animals [1]. However, *T. pyogenes* is also an important opportunistic pathogen that causes mastitis, abortion and a variety of diverse pyogenic infections in livestock, including cattle, sheep, goats, horses, and pigs [2–4]. In cattle, *T. pyogenes* appears to be responsible for infections of the reproductive tract [5] and the mammary gland [6], as well as cases of pneumonia and liver abscessation of large and small ruminants [7]. In swine, *T. pyogenes* is well known as a causative agent of different types of inflammation in various organs including the lung, heart, joints, mammary glands, and in the reproductive tract [8, 9]. Furthermore, *T. pyogenes* could be found in companion animals [4]. One of the first reported cases in companion animals was an otitis externa detected in a cat and cystitis in a dog [10]. More recently, Wareth et al. [11] described a co-infection case of *T. pyogenes* with *Brucella abortus* in a cat and dog. Additionally, various wildlife animals could harbour *T. pyogenes* [3]. In 2010, Ülbegi-Mohyla et al. [12] characterized two *T. pyogenes* strains isolated from a bearded dragon and a gecko. Additionally, *T. pyogenes* infections were reported from a bison and from camels [13, 14], from goitered gazelles [15] and from a white-tailed deer [16]. Likewise, some other sporadic cases of infectious diseases associated with *T. pyogenes* were described in a galago [17], in gray slender lorises [18, 19] and in a eurasian lynx [20].

Besides conventional bacteriological methods for identifying *T. pyogenes* isolates, other new, fast and reliable techniques were described and utilized in this study: matrix-assisted laser desorption/ionization time-of-flight mass spectrometry (MALDI-TOF MS) [20–23], a loop-mediated isothermal amplification (LAMP) assay [24] and 16S rRNA gene sequencing [25, 26].

The present study was designed to identify and further characterize *T. pyogenes* isolated from wildlife animals phenotypically and genotypically. To the best of our knowledge, the present study provides a first detailed description of *T. pyogenes* recovered from an okapi and a royal python.

## Case presentation

As part of routine examination and diagnostics performed on zoo animals at Frankfurt Zoo (Frankfurt am Main, Germany) in 2019, *T. pyogenes* 24398 was isolated from a vaginal discharge of an okapi (*Okapia johnstoni*) in high numbers (+++), together with *Enterobacter cloacae* (+) and *Pasteurella* spp. (+). The initial bacteriology analysis for *T. pyogenes* 24398 was performed at Hessian State Laboratory (LHL) Gießen, Germany. As a result of post-mortem examination conducted in 2017, *T. pyogenes* 171003246 was recovered in low numbers (+) from a kidney of a seven-year-old female royal python (*Python regius*). The python was found dead in a bird park in Hesse (Germany) and was 107 cm in length and weighted 1.23 kg. In addition, *Escherichia coli* (+), α-hemolytic streptococci (+), *Corynebacterium* spp. (+) and *Clostridium sardiniense* (+) were cultured from the python specimen. The post-mortem analysis of the royal python revealed a good body condition and in the throat and head area a 15 cm lung edema and swelling, possibly caused by traumatic reasons. The post-mortem examination and the initial bacteriology analysis were also performed at Hessian State Laboratory. Both *T. pyogenes* strains were further investigated phenotypically and genotypically.

### Phenotypic characterization

A phenotypic characterization was performed using conventional cultural and biochemical assays as previously described [12, 18, 20, 27] and the API-Coryne test System (BioMérieux, Nürtingen, Germany) in accordance with the manufacturer’s instructions. Furthermore, the bacterial isolates were identified by MALDI-TOF MS using a Microflex LT (Bruker Daltonik GmbH, Bremen, Germany) instrument and MBT Compass Explorer 4.1 software (Bruker Daltonik GmbH). Sample preparation was carried out in accordance with the manufacturer’s instructions using the direct transfer method. Briefly, one microbial colony was first smeared in duplicate onto spots of the MALDI target MSP 96 target (MicroScout Target plate; Bruker Daltonik GmbH) with sterile toothpicks. The air-dried bacteria were overlaid with 1 µl of an α-cyan 4-hydroxycinnamic acid matrix solution (HCCA, in 50% acetonitrile and 2.5% trifluoroacetic acid in pure water) followed by drying and loading into the mass spectrometer.

### Genotypic properties

The genomic DNA of both isolates and the type strains *T. pyogenes* DSM 20630^T^ (pig), *T. abortisuis* DSM 19515^T^ (placenta of sow after abortion), *T. bernardiae* DSM 9152^T^ (human blood) and *T. bonasi* DSM 17163^T^ (european bison) were extracted using the DNeasy blood and tissue kit (Qiagen GmbH, Hilden, Germany), in accordance with the manufacturer’s instructions. The concentration and purity of DNA were measured by utilizing a Nano Drop spectrophotometer (ND1000; Thermo Fisher Scientific GmbH, Dreieich, Germany).

The detection of gene *cpn*60 of *T. pyogenes* was performed using a previously designed loop-mediated isothermal amplification (LAMP) assay [24] with a portable real-time fluorometer (Genie II®, OptiGene Ltd, Horsham, UK) and the reference strains *T. pyogenes* DSM 20630^T^, *T. abortisuis* DSM 19515^T^, *T. bernardiae* DSM 9152^T^ and *T. bonasi* DSM 17163^T^.

Both *T. pyogenes* isolates were also evaluated by PCR for the presence of five genomic targets: 16S rRNA gene (16S), 16S-23S rDNA intergenic spacer region (ISR), the β-subunit of bacterial RNA polymerase encoding gene *rpo*B, the elongation factor tu encoding gene *tuf*, and pyolysin encoding gene *plo*. The sequence of the oligonucleotide primers and PCR conditions were previously described by Hassan et al. [25], Ülbegi-Mohyla et al. [12], Hijazin et al. [27], Eisenberg et al. [18], Wickhorst et al. [28], Wickhorst et al. [23], Alssahen et al. [20].

The PCR products were purified and sequenced by Eurofins Umwelt Nord GmbH (Göttingen, Germany). The obtained sequences were analyzed via the cluster method of the MegAlign program (DNASTAR Inc., ver. 15, Madison, WI, USA) by comparing with the nucleotide sequences of 16S rRNA, ISR, *rpo*B, *tuf* and *plo* from different *Trueperella* reference strains. Moreover, the resulting amino acid sequences of pyolysin of both *T. pyogenes* isolates were compared with the respective sequences of pyolysin of *T. pyogenes* DSM 20630^T^, closely related pore-forming toxins of genus *Arcanobacterium* and with other bacterial pore-forming toxins. All the nucleotide and amino acid sequences were obtained from the NCBI GenBank.

## Discussion and Conclusion

Both *T. pyogenes* strains investigated in the present study showed a narrow zone of complete hemolysis on 5% sheep blood agar and CAMP-like reactions in the staphylococcal β-hemolysin zone with *Rhodococcus hoagii* as an indicator strain. The conventional biochemical properties and the results of the commercial identification system revealed almost identical results to previously investigated *T. pyogenes* of various origins and *T. pyogenes* DSM 20630^T^ [12, 18, 20, 27] (Table. 1). The *T. pyogenes* isolates yielded positive reactions for pyrrolidonyl arylamidase, alkaline phosphatase, β-glucuronidase, β-galactosidase, α-glucosidase and N-acetyl-β-glucosaminidase and negative reactions for nitrate reduction and pyrazinamidase. Additionally, the isolates hydrolyzed gelatine, but not esculin and urea. The isolates also fermented D-glucose, D-ribose, D-xylose, D-maltose, D-lactose and glycogen, but not D-mannitol. *T. pyogenes* 24398 fermented D-saccharose; however, *T. pyogenes* 171003246 was D-saccharose negative. In addition, both isolates showed a negative catalase reaction and a positive reaction on Löffler agar (Table 1). A postitive reaction on Löffler agar is typical for *T. pyogenes* and widely used for phenotypic identification of this species [2, 18].

**Table 1:** Biochemical properties of *T. pyogenes* 24398 (okapi), *T. pyogenes* 171003246 (royal python) and type strain *T. pyogenes* DSM 20630^T^

Biochemical properties	*T. pyogenes* 24398 (okapi)	*T. pyogenes* 171003246 (royal python)	*T. pyogenes*^a^ DSM 20630^T^
Haemolysis on SBA^b^	+	+	+
CAMP-like hemolytic reaction with^c^:			
*Staphylococcus aureus*	+	+	+
*Streptococcus agalactiae*	−	−	−
*Rhodococcus hoagii*	+	+	+
Reverse CAMP reaction	−	−	−
Nitrate reduction	−	−	−
Pyrazinamidase	−	−	−
Pyrrolidonyl Arylamidase	+	+	+
Alkaline phosphatase	+	+	+
β-Glucuronidase (β-GUR)	+	+	+
β-Galactosidase (β-GAL)	+	+	+
훼α-Glucosidase (훼α-GLU)	+	+	+
N-Acetyl-β-glucosaminidase (β-NAG)	+	+	+
Esculin (β-glucosidase)	−	−	−
Urease	−	−	−
Gelatine	+	+	+
**Fermentation of**:			
Glucose	+	+	+
Ribose	+	+	+
Xylose	+	+	+
Mannitol	−	−	−
Maltose	+	+	+
Lactose	+	+	+
Saccharose	−	+	−
Glycogen	+	+	+
Catalase	−	−	−
Serolysis on Loeffler agar	+	+	+
Identification % according to API-Coryne test System	99.9	99.9	99.9

^a^Results taken from (Ülbegi-Mohyla et al. 2010 [12]; Hijazin et al. 2011 [27]; Eisenberg et al. 2012 [18]; Alssahen et al. 2020 [20])

^b^SBA: Sheep Blood Agar

^c^synergistic or reverse CAMP-like reaction with indicator strains

+: positive reaction, −: negative reaction, ^**T**^:type strain

Moreover, MALDI-TOF MS identified *T. pyogenes* 24398 and *T. pyogenes* 171003246 with log score values of 2.35 and 2.29 for the first hit and log score values of 2.28 and 1.9 for the second hit, respectively (data not shown). These log score values confirmed, in accordance with the current decision rules of the manufacturer, the species designation. Comparable to the present results, MALDI-TOF MS had already been shown to be a rapid and reliable technique for identifying bacteria of genera *Arcanobacterium* and *Trueperella*, including *T. pyogenes* [20, 21].

**Fig. 1:**
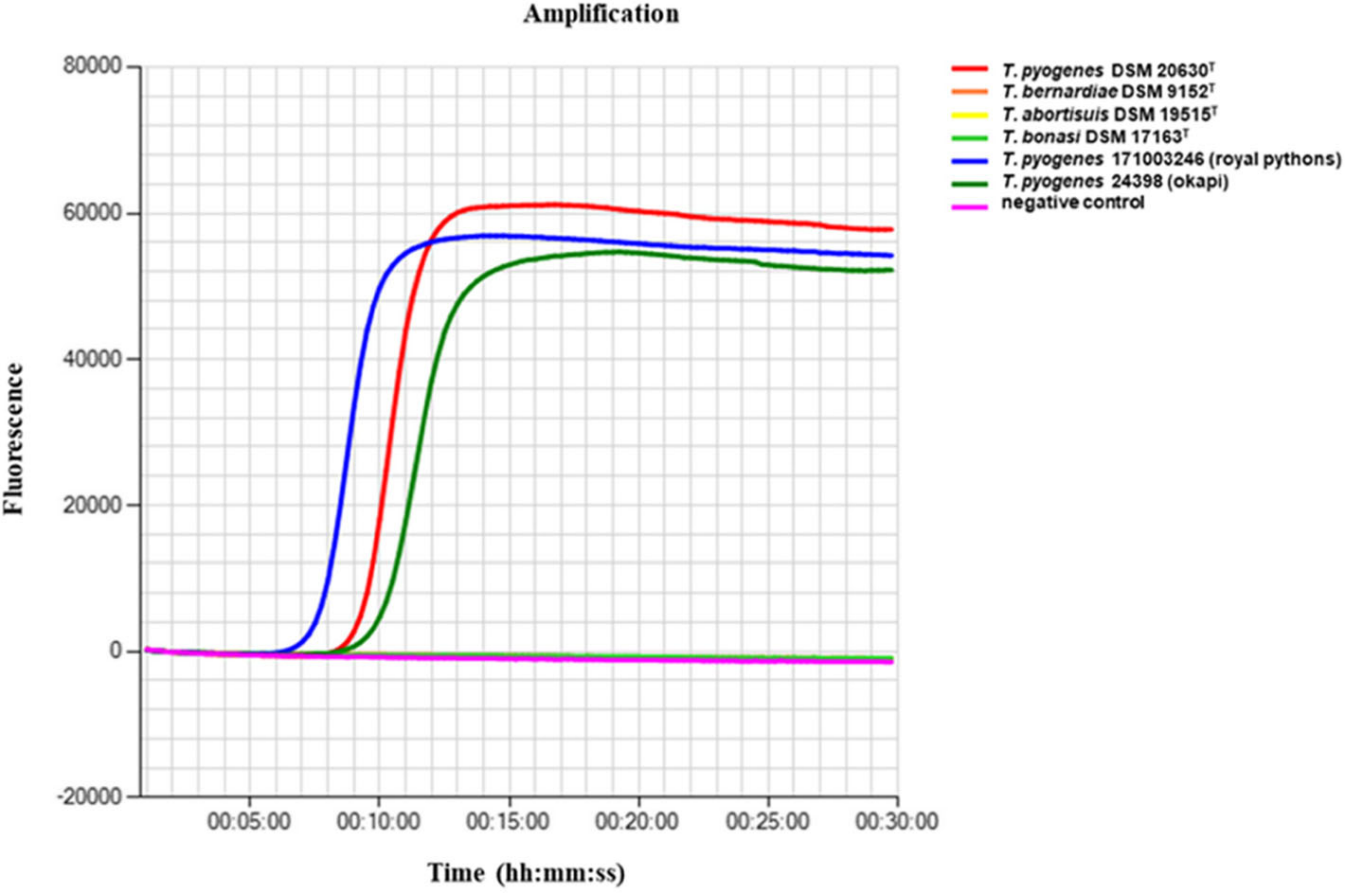
Positive LAMP assay of* T. pyogenes *24398 (okapi), *T. pyogenes *171003246 (royalpython),* T. pyogenes *DSM 20630^T^, the LAMP negative control strains *T. abortisuis* DSM 19515^T^,* T. bernardiae*DSM 9152^T^and* T. bonasi* DSM 17163^T^, and a negative control

The previously described *cpn*60-specific LAMP assay could successfully be used to identify the species-specific gene *cpn*60 of *T. pyogenes* 24,398 and *T. pyogenes* 171,003,246 in the present investigation. This was comparable to the LAMP assay for detecting gene *cpn*60 of the previously described *T. pyogenes* of various origins [24], a *T. pyogenes* strain isolated from an adult roebuck (*Capreolus capreolus*) [23], and a *T. pyogenes* strain isolated from a eurasian lynx (*Lynx lynx*) [20]. The results of the *cpn*60 LAMP assay are shown in Fig. 1; Table 2.

**Table 2:** Results of LAMP including detection time and annealing temperature of the tested isolate, positive and negative control

Sample ID	*T. pyogenes* 24398	*T. pyogenes* 171003246	*T. pyogenes* DSM 20630^T^	*T. abortisuis* DSM 19515^T^	*T. bernardiae* DSM 9152^T^	*T. bonasi* DSM 17163^T^	HPLC water and Master mix
Result	+ve	+ve	+ve	-ve	-ve	-ve	-ve
Detection time hh:mm:ss	00:11:00	00:08:30	00:10:00	0	0	0	0
Annealing	89.6	89.4	89.9	0	0	0	0

+ve: Positive, -ve: Negative, ^**T**^:type strain

**Fig. 2:**
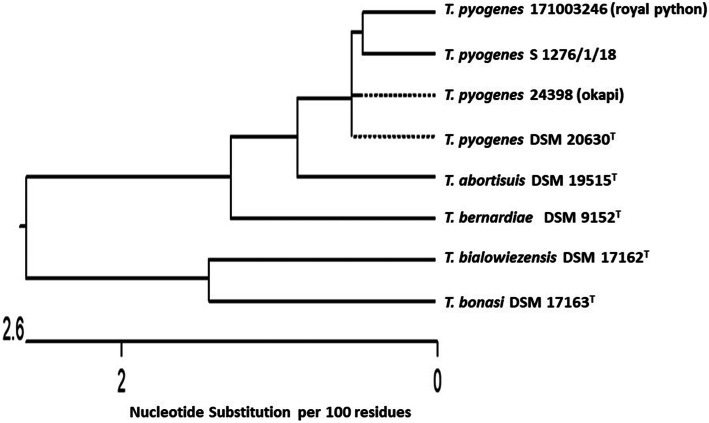
Phylogenetic analysis based on nucleotide sequences of 16S rRNA gene of the investigated *T. pyogenes* 24398 and *T. pyogenes* 171003246 isolated from okapi and royal python compared with the type strain *T. pyogenes* DSM 20630^T^ and *T. pyogenes* S 1276/1/18 isolated from a eurasian lynx, *T. abortisuis* DSM 19515^T^, *T. bernardiae* DSM 9152^T^, *T. bialowiezensis* DSM 17162^T^, and *T. bonasi* DSM 17163^T^

The oligonucleotide primers, 16SUNI-L and 16SUNI-R, were used for amplifying of 16S rRNA gene of the investigated *T*. *pyogenes* isolates. The nucleotide sequence data of *T. pyogenes* 24398 (GenBank accession numbers: MN946520) and *T. pyogenes* 171003246 (MN712476) were compared with type strain *T. pyogenes* DSM 20630^T^ (AAC45754) and with the previously described strain *T. pyogenes* S 1276/1/18 isolated from a eurasian lynx (MN135984), *T. abortisuis* DSM 19515^T^ (FN667628), *T. bernardiae* DSM 9152^T^ (X79224), *T. bialowiezensis* DSM 17162^T^ (EU194569), and *T. bonasi* DSM 17163^T^ (EU194570). The nucleotide sequence data of *T. pyogenes* 24398 and *T. pyogenes* 171003246 revealed a sequence homology of 98.9% among both strains, a sequence homology of 99.5% and 98.7% with *T. pyogenes* DSM 20,630^T^, and a sequence homology of 99.9% and 99.1% with *T. pyogenes* S1276/1/18, respectively. The control strains of genus *Trueperella* yielded a sequence homology to both *T. pyogenes* isolates ≤ 98.7% (Fig. 2).

Both strains *T. pyogenes* 24398 and *T. pyogenes* 171003246 were further identified by sequencing ISR, the genes *tuf* and *rpo*B and the putative virulence factor pyolysin encoding gene *plo*. *T. pyogenes* 24398 and *T. pyogenes* 171003246 showed sequence similarities of ISR (MN947249, MN724920) of 99.8% and 98.9% with *T. pyogenes* DSM 20630^T^ (EU194563) and 100% and 99.8% with *T. pyogenes* S 1276/1/18 (MN164031), respectively with 98.5% identity between both strains. The additionally investigated gene *tuf* (MN956808, MN741111) showed a sequence similarity of 99.6% and 99.7% with *T. pyogenes* DSM 20630^T^ (HG941716), and 99.6% and 99.7% with *T. pyogenes* S 1276/1/18 (MN163266), respectively; gene *rpo*B (MN956807, MN741109), a sequence similarity of 99.8% and 98.3% with *T. pyogenes* DSM 20630^T^ (FN550375), and 98.8% and 98.3% with *T. pyogenes* S 1276/1/18 (MN163265), respectively, and gene *plo* (MN956806, MN741110), a sequence similarity of 99.5% with *T. pyogenes* DSM 20630^T^ (U84782) for both isolates, and 99.1% with *T. pyogenes* S 1276/1/18 (MN163264) for both isolates.

**Fig. 3:**
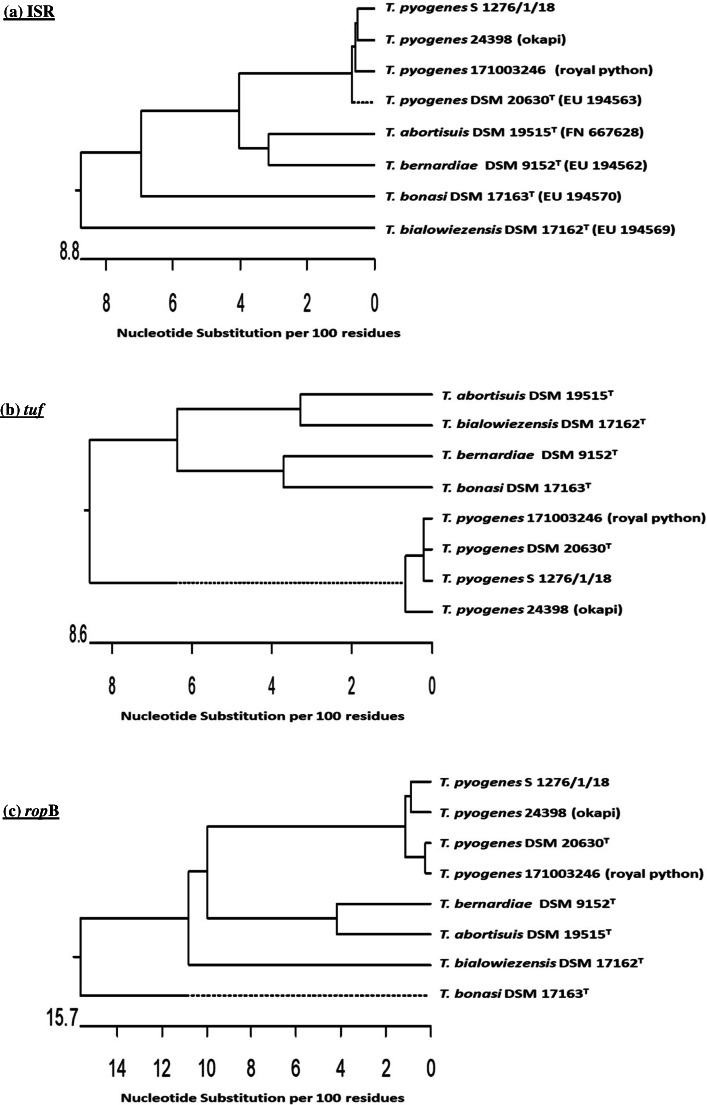
Phylogenetic analyses based on ISR (**a**), *tuf* (**b**) and *rop*B (**c**) nucleotide sequences of the investigated *T. pyogenes* 24398 and *T. pyogenes* 171003246 and *T. pyogenes* S 1276/1/18 isolated from a eurasian lynx and the control strains, *T. pyogenes* DSM 20630^T^, *T. abortisuis* DSM 19515^T^, *T. bernardiae* DSM 9152^T^, *T. bialowiezensis* DSM 17162^T^, and* T. bonasi* DSM 17163^T^

Dendrograms of the ISR, *tuf* and *rpo*B genes are presented in Fig. 3.

**Fig. 4:**
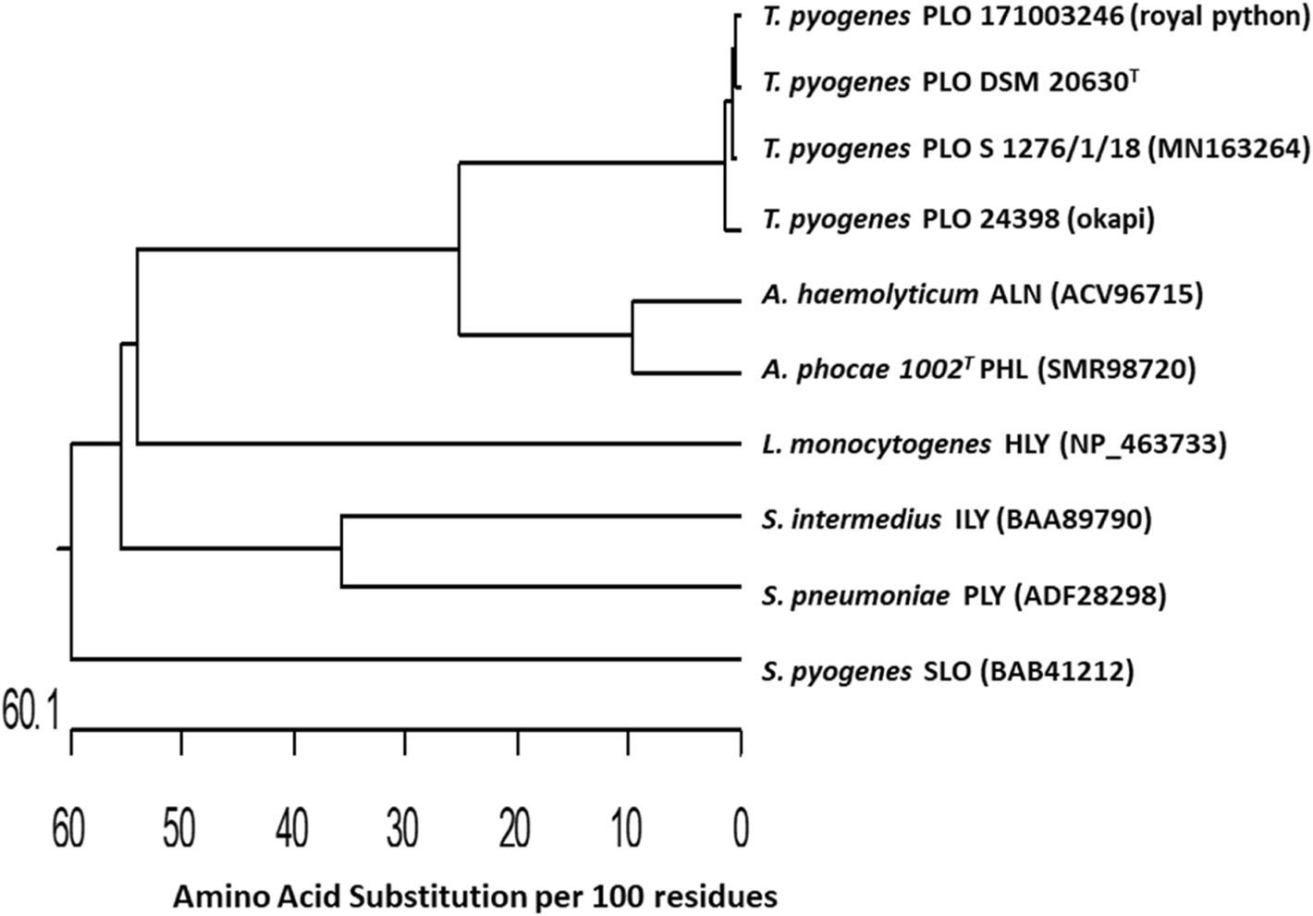
Phylogenetic relationships among amino acid sequences of PLO of the investigated *T. pyogenes* 24398 and *T. pyogenes* 171003246, PLO of type strain *T. pyogenes* 20630^T^, *T. pyogenes* S 1276/1/18 isolated from a eurasian lynx, ALN of *A. haemolyticum*, PHL of *A. phocae*, HLY of *L. monocytogenes*, PLY of *S. pneumoniae*

A phylogenetic analysis of the amino acid sequences of pyolysin (PLO) encoded by gene *plo* of *T. pyogenes* 24398 (MN956806), and *T. pyogenes* 171003246 (MN741110) PLO of type strain *T. pyogenes* DSM 20630^T^ (AAC45754), PLO of *T. pyogenes* S 1276/1/18 (MN163264), arcanolysin (ALN) of *Arcanobacterium haemolyticum* (ACV96715), phocaelysin (PHL) of *Arcanobacterium phocae* 10002^T^ (SMR98720), listeriolysin O (HLY) of *Listeria monocytogenes* (NP_463733), intermedilysin (ILY) of *Streptococcus intermedius* (BAA89790), pneumolysin (PLY) of *Streptococcus pneumoniae* (ADF28298) and streptolysin O (SLO) of *Streptococcus pyogenes* (BAB41212). The results showed an amino acid similarity of 99.5% for both *T. pyogenes* 24,398 and *T. pyogenes* 171003246 with PLO of *T. pyogenes* DSM 20630^T^ and 99.1% with PLO of *T. pyogenes* S 1276/1/18 (Fig. 4).

*T. pyogenes* 24398 was isolated in high numbers from vaginal discharge of an okapi and seems to be responsible for the infectious process; *T. pyogenes* 171003246 was isolated from a non-infectious process of a royal python suffering from a throat swelling, possibly caused by trauma. Both *T. pyogenes* isolates were identified by a biochemical test, LAMP and MALDI-TOF MS. The genomic targets of the two isolates, 16S rRNA gene, ISR, *tuf*, *rpo*B and *plo* were sequenced and compared to the respective targets of reference and other strains. Thus, the report is the first to provide a detailed characterization of *T. pyogenes* strains of these origin.

## Data Availability

The datasets generated during the current study are available in the NBCI GenBank repository, under the accession number: MN741109, MN741110, MN741111, MN956806, MN956807 and MN956808.

## Abbreviations

MALDI-TOF MS: Matrix-assisted laser desorption/ionization time-of-flight mass spectrometry
LAMP: Loop-mediated isothermal amplification
rRNA: Ribosomal RNA
ISR: 16S-23S rDNA intergenic spacer region
CAMP test: Christie-Atkins-Munch-Peterson-test
DSM: German Collection of Microorganisms
ALN: Arcanolysin
PHL: Phocaelysin
HLY: Listeriolysin O
ILY: Intermedilysin
PLY: Pneumolysin
SLO: Streptolysin O
